# Genetic and Tissue Engineering Approaches to Modeling the Mechanics of Human Heart Failure for Drug Discovery

**DOI:** 10.3389/fcvm.2018.00120

**Published:** 2018-09-19

**Authors:** Michael J. Greenberg, Neil J. Daily, Ann Wang, Michael K. Conway, Tetsuro Wakatsuki

**Affiliations:** ^1^Department of Biochemistry and Molecular Biophysics, Washington University School of Medicine, St. Louis, MO, United States; ^2^InvivoSciences Inc., Madison, WI, United States

**Keywords:** heart failure, tissue engineering, length-tension relationship, gene editing, human induced pluripotent stem cells, high-throughput screening, rare heart disease, drug discovery

## Abstract

Heart failure is the leading cause of death in the western world and as such, there is a great need for new therapies. Heart failure has a variable presentation in patients and a complex etiology; however, it is fundamentally a condition that affects the mechanics of cardiac contraction, preventing the heart from generating sufficient cardiac output under normal operating pressures. One of the major issues hindering the development of new therapies has been difficulties in developing appropriate *in vitro* model systems of human heart failure that recapitulate the essential changes in cardiac mechanics seen in the disease. Recent advances in stem cell technologies, genetic engineering, and tissue engineering have the potential to revolutionize our ability to model and study heart failure *in vitro*. Here, we review how these technologies are being applied to develop personalized models of heart failure and discover novel therapeutics.

Heart failure (HF) is the leading cause of death in the United States, accounting for 1 in 9 deaths that occur each year and over $30 billion in annual health care costs (1). Chronic HF affects ~2% of the population <60 years old and >10% of adults >75 years old (2). HF is characterized by the inability of the heart to generate sufficient cardiac output to effectively pump blood to the body under normal physiological pressures. Clinically, HF patients are classified by their ejection fraction (i.e., the fraction of blood that is pumped out of the ventricles with each beat). HF with reduced ejection fraction (HFrEF) can be caused by several conditions, such as valvular disease, myocardial infarction, and some genetic cardiomyopathies (described in more detail below). Several treatment options are available for HFrEF, including ACE inhibitors, beta blockers, and implantable devices (3); however, many of these therapeutic options have significant side effects, including tachycardia and arrhythmia (4). HF with preserved ejection fraction (HFpEF) is characterized by diastolic dysfunction, such as impaired filling due to fibrotic stiffening of the ventricular wall, but a normal ejection fraction. HFpEF can be caused by several conditions including chronic hypertension, aging, metabolic syndrome, and several genetic cardiomyopathies. Despite the number of efforts in clinical trials to date, no efficacious therapies have been identified for HFpEF (5–8).

Even with the best treatments available, there are high rates of mortality and morbidity with both HFrEF and HFpEF (9). This is partly due to our lack of mechanistic understanding of the disease pathogenesis (10) and the lack of an appropriate *in vitro* model system that can recapitulate relevant aspects of cardiac mechanics with sufficient throughput for drug discovery. Here, we review several recent advances in the fields of genetic and tissue engineering that have made it possible to model aspects of these diseases *in vitro*, and we discuss the potential applications of these technologies to drug discovery and personalized medicine.

## Current challenges in modeling human heart diseases

Studying cardiovascular disease *in vitro* comes with several challenges. First, cardiac physiology is tightly regulated in whole organisms by complex neuronal and hormonal feedback systems (11–14). Perturbations affecting cardiac function can lead to both short-term adaptations of the heart (e.g., increased heart rate, length-dependent changes in contractility, increases in the phosphorylation of sarcomeric proteins such as troponin-I or titin, and force-induced changes on actomyosin contractility), as well as long-term adaptations (e.g., cellular reorganization, cardiac tissue remodeling, activation of fibroblasts, and changes in gene expression). Understanding the disease pathogenesis and the development of novel therapeutics requires tools for studying the disease phenotypes across multiple scales of organization, ranging from the level of single molecules to whole organisms.

Another major challenge to modeling HF is the heterogeneity in the prognosis and presentation of HF in patients (15). As described earlier, HF patients are typically characterized by ejection fraction (i.e., their symptoms), but there are multiple underlying conditions that cause HF. For example, non-genetic HF can be initiated by myocardial injury (e.g., myocardial infarction) (16), valvular disease (17), or as a side effect of some chemotherapies (18). There are also several forms of genetic heart disease that can lead to heart failure (19–22). Familial hypertrophic (HCM) and dilated (DCM) cardiomyopathies are primarily caused by mutations in proteins that regulate cardiac muscle power output. HCM is characterized by thickening of the ventricular wall, fibrosis, and myocyte disarray. It has an estimated prevalence of 1 in 500 people, and it is the leading cause of sudden cardiac death in people under 30 years old (23). Familial DCM is a closely related disease that is also strongly associated with sudden death, and it is a significant cause of HF (24). DCM is characterized by dilation of the myocardial wall, and it is often accompanied by necrosis and fibrosis. Even though these forms of genetic heart disease are relatively common, the clinical presentations and the prognoses of HCM and DCM are highly variable and depend on the exact pathogenic mutation. To date, hundreds of mutations have been associated with these diseases (19, 23). Point mutations within the same molecule can lead to either HCM or DCM, with the phenotype depending on the specific site of the mutation (25, 26). Therefore, when modeling these genetic diseases, it is perhaps more useful to think of these conditions as collections of rare diseases with a common presentation. As such, the design of therapeutics presents itself as an opportunity for personalized treatment (i.e., precision medicine) (27).

Several *in vitro* model systems have been developed to address the challenges associated with modeling HF, each with its own set of advantages and drawbacks. The choice of model system is dictated by the specific questions being asked. For example, in many patients with HCM or DCM, point mutations in sarcomeric proteins at the molecular level are the initial insults that lead to tissue remodeling in the disease. Understanding these diseases requires a molecular knowledge of the specific defects caused by the mutations (25), and excellent experiments using purified and/or expressed proteins have led to the development of several drugs that are currently in clinical or preclinical trials (28–31). While these experiments are needed to dissect the initial molecular insults that lead to the disease phenotype, they have several caveats. First, the majority of biochemical studies are conducted in the absence of load, and it has been shown that mechanical forces can change the kinetics (and thus functional properties) of proteins, including cardiac myosin (32–39). This is important since proteins in the heart experience both internally and externally generated forces during contraction, and aberrant forces are a primary driver of cardiomyopathies (40). In fact, for some HCM mutations, the molecular disease phenotype only becomes apparent under load (39), and thus one must consider the mechanobiology of the heart when studying these diseases. Second, changes in contractility at the molecular level *in vitro* are not necessarily predictive of how the disease affects contractility in cells or tissues. For example, the first mutation identified to cause HCM, R403Q in MYH7 (20), shows conflicting results at the molecular level (41–43) that do not necessarily correspond to the phenotype in mice (44–46). Moreover, some forms of genetic HF are due to haploinsufficiency rather than direct changes in protein function (47).

Another approach that has greatly furthered the understanding of both genetic and non-genetic HF is the use of transgenic mouse models for physiological and biochemical studies [e.g., (42, 44, 48–55)]. This system allows for control of the genetic environment and physiological studies. However, mouse hearts have very different physiology than human hearts. For example, mouse hearts can beat ~600 times per minute while human hearts beat ~60 times per min. To beat this quickly, mouse hearts have some different ion channels [e.g., different subunits for the K-ATP channel (56) and different I_Kr_ channels (57)] that define their action potentials, different machinery for handling calcium, and different myosin isoforms with disparate kinetics that drive contractility (58–60). Therefore, transgenic mouse models do not always recapitulate the human disease phenotype and pharmacological response (44, 46, 48, 61–64). Also, mouse hearts lack the hERG channel. Many drugs, both cardiac-specific and nonspecific, can bind to this protein, leading to cardiotoxicity and arrhythmias in humans, despite having no effects in mice. This missed cardiotoxicity is one of the reasons that drugs designed based on mouse studies fail in clinical trials (65, 66).

Tissue obtained from patients (67) gives unique insights into the disease pathogenesis that cannot be recapitulated in other systems. However, it is difficult to obtain human tissue, and the disease presentation is often complicated by the patient's genetic background and medical history. Moreover, human tissues are usually obtained from patients whose hearts have undergone major remodeling and changes in gene expression in response to the disease. As such, it is not necessarily a good model system for studying how the initial insult of the mutation affects cardiac functions including contractility. Also, in the case of genetic heart disease, it is difficult to obtain sufficient tissue with a given genotype for well controlled drug testing. Moreover, it is challenging to get appropriate control tissue, since differences in the genetic background and patient history can affect the observed phenotype (68).

## Human pluripotent stem cell derived cardiomyocytes as models of disease

Recent advances in stem cell and genome editing technologies have led to the development of human pluripotent stem cell (hPSC)-based models of genetic human cardiac diseases. A critical advance was the derivation of human embryonic stem cell lines (69, 70) and their subsequent differentiation to a cardiomyocyte lineage (71). These early studies, which relied on embryoid body formation, had a very low differentiation efficiency (<1%) (71). Several methods have been developed to increase the efficiency of differentiation of stem cells to hPSC-CMs in both embryoid bodies (72, 73) and adherent monolayers of cells (74, 75). One widely used method, where WNT signaling is initially stimulated to promote mesoderm formation and then repressed to induce a cardiomyocyte lineage (74), can produce >90% hPSC-CMs (75, 76).

One difficulty with hPSC-CMs is that the differentiation methods produce a mixture of atrial, ventricular, pacemaker, and non-myocyte cells; although techniques have been developed recently to promote differentiation toward a specific cardiac lineage (77–80) and to eliminate non-myocytes from the cell culture (81). An additional challenge with hPSC-CMs is that they are developmentally immature (82, 83). This immaturity can be seen in several aspects of the cell physiology, including the ratio of alpha (MYH6) to beta (MYH7) cardiac myosin, the shape of the action potential, the absence of t-tubules, and the orientation of sarcomeres within the cardiomyocyte (84, 85). Although hPSC-CMs are developmentally immature (86, 87), they are an ideal system for studying the early disease pathogenesis, before the heart undergoes many of the adaptations seen in older patients. Moreover, several approaches have been used to engineer more mature phenotypes in hPSC-CMs, including electrical pacing (88–91), addition of growth hormones or fatty acids (82, 92), providing mechanical or geometric cues that mimic the organization of the heart (93–96), and providing stretch/mechanical resistance (97–100). hPSC-CMs can also be matured through incorporation into 3D engineered tissues.

Several groups have derived stem cells from patient samples [e.g., (47, 101–103)] and then differentiated these cells to hPSC-CMs. These studies have shown that it is possible to recapitulate aspects of cardiac disease using these cells. Recent advances in genetic engineering, such as the application of the CRISPR/Cas9 system (104, 105), have opened the door to studying genetic forms of heart failure and the role of genetic modifiers in disease without the need for patient heart tissue. These tools have been harnessed to introduce disease-causing mutations into hPSCs and then study their phenotypes [e.g., (47, 103)]. The genome editing approach has the advantage that the mutant and WT lines are isogenic except for the pathogenic mutation. This is important since cardiomyopathies often show incomplete penetrance, and the disease presentation can vary depending on the genetic background (68, 106). A disadvantage to using genetic engineering of healthy cells instead of patient cells is the inability to directly correlate changes *in vitro* with relevant clinical data of cardiac function *in vivo*. Moreover, the disease presentation depends on the genetic background, and therefore, the presentation in a control cell line could differ from the presentation in a patient. However, it is possible to take cells from a patient with the disease and then fix the genetic mutation to generate genetically matched control cells (107). This later approach has the advantage that it enables the collection of *in vivo* clinical data from the patient and then the correlation of these parameters with properly controlled measurements *in vitro*.

## Human engineered heart tissues

The human heart has a complex three-dimensional structure composed of many cell types including cardiomyocytes, fibroblasts, macrophages, and endothelial cells. The cardiomyocytes interact with the other cell types, and these other cells can modulate the contractile and electrophysiological properties of cardiomyocytes (108–113). These cells are organized within the extracellular matrix to give rise to distinct regions within the heart with specific functions (e.g., sinoatrial node, ventricular wall, and papillary muscles). Moreover, these cells can be mechanically and electrically coupled, and the mechanical environment can affect the electrophysiological properties of these cells (114). The cells in the heart are thus subjected to an array of mechanical, chemical, and electrical signals that can affect their function. Generating *in vitro* models of heart disease that faithfully recapitulate cardiac dysfunction will require consideration of these complexities.

To recapitulate many of these aspects of cardiac functions *in vitro*, 3D engineered heart tissues (EHT) were first created more than two decades ago using cardiomyocytes isolated from chicken embryos (115). Since then, the successful fabrication of EHTs with hPSC-CMs has significantly advanced our ability to model human heart diseases *in vitro*, and these tissues faithfully recapitulate many features of the clinical disease phenotypes [e.g., (47, 116–118)]. In addition, miniaturization of the EHTs has enabled mass-production of EHTs for higher throughput assays (111–113, 119) The hPSC-CMs in EHTs exhibit more mature phenotypes than those grown in 2D culture, showing more normal sodium currents (120), organized sarcomeric arrangement (121), and improved mitochondrial function (88).

The 3D environment of EHTs allows researchers to control and recapitulate mechanical homeostasis unique to the heart (122, 123). Scaffold-free 3D spheroid tissue models have advantages for simple high-throughput assays, but they lack the mechanobiological cues necessary for tissue maturation and organization (124, 125). EHTs formed using parallel wires (89, 126–128), parallel posts (98, 129–131), or sheets (132) can provide an improved mechanical microenvironment for EHT development. Mechanically stretching EHTs improves the maturation of myocytes (98, 129, 133–135) and can increase cellular alignment (136–138). Combined electrical and mechanical conditioning of EHTs has shown promising results for cardiac tissue maturation (88, 137, 139).

While EHTs are powerful tools for studying heart disease, there are various limitations that must be considered. Since EHTs are fabricated in 3D, many cells are needed to fabricate a single sample. Therefore, the costs and times required to produce EHTs are generally higher than those of 2D cell culture. Additionally, the production of EHTs requires the quality control of many more parameters due to their complexity. For example, the differentiation efficiency of stem cells to hPSC-CMs, the number of stromal cells added to the tissue, and the formation of defined extracellular matrices are very important for reproducibility. Moreover, care must be taken when selecting an appropriate culture media, since supplements in the medium can affect certain cell types in the EHT and modulate the activity of enzymes that remodel the extracellular matrix (ECM). While cardiac tissues can be formed without adding any exogenous ECMs components using cell sheet technology (140), most EHTs use exogenously added ECMs. While collagen and fibrin are the most popular choices for the ECMs in EHTs, their hydrogel properties can be different depending on their methods of preparation (141, 142). Other ECM components such as basement membrane proteins can be doped into the base ECM to mimic the composition of ECMs in the heart. The field will benefit from continued examination of how different ECM compositions influence the physiological properties of EHTs, especially with regard to changes in the ECM associated with HF.

## Measurement of cardiac mechanics using human engineered heart tissues

To date, many different platforms for EHTs have been developed. These platforms have been tailored for specific applications, with systems that excel at modeling different aspects of the heart, including vascularization, microcirculation, cardiomyocyte maturity, structure, calcium handling, and contractility (47, 88, 119, 143–146). The selection of the appropriate EHT system will depend on the specific questions being asked.

**Figure 1:**
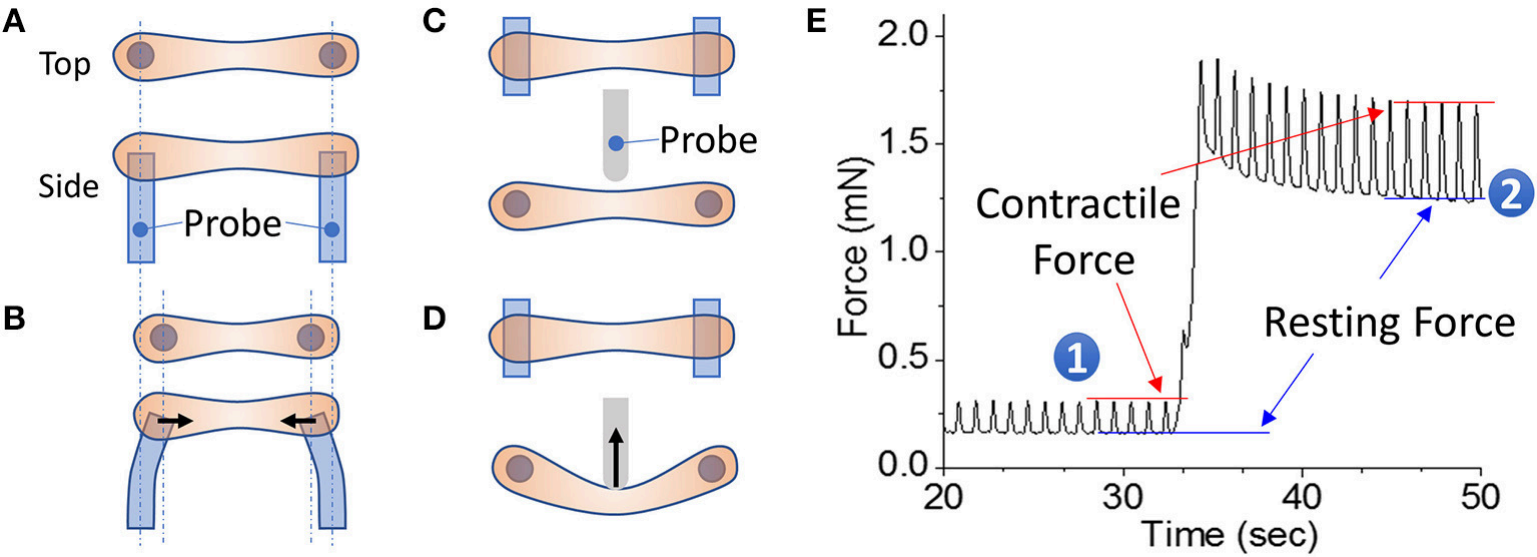
EHT strips formed in a 96-well format for phenotyping assays. **(A,B)** Example of a passive force system in which an EHT strip is formed between two parallel posts. The force generated during contraction can be monitored by the deflection of the posts. **(C,D)** Example of an active force system in which an EHT strip is formed between two parallel posts and then the tissue can be stretched using a probe that pushes on the side of the tissue. In this setup, the force is measured by a strain gauge in the probe. **(E)** Measurement of cardiac forces at two different muscle lengths (1 and 2). The peak and bottom of the cardiac twitch force profiles correspond to the contractile and resting forces, respectively. Note that stretching the EHT causes an increase in the contractile force, as would be expected from the Frank-Starling relationship.

In both HFpEF and HFrEF, the mechanics of the heart are altered; and therefore, when modeling HF *in vitro*, it is desirable to be able to examine the effects of the disease on cardiac contractility. In most EHT systems capable of modeling cardiac contractility, an EHT in a hydrogel is formed between two posts and the contractility of the tissue is measured using a transducer (Figure 1). Human hPSC-CMs and human cardiac fibroblasts in the EHT remodel the hydrogel to form cardiac tissue strips (or sheets), where the cells are aligned perpendicular to the parallel posts. The transducers used in most of these systems measure the force of contraction by monitoring the deflection of the posts. The deflection can be measured using electronic strain gauges or using optical detection of the post position.

EHT systems for measuring contractility can be broadly divided into passive and active force systems, depending on whether the tissue can be actively stretched in real time during an experiment or only passively monitored. Passive force systems are easier and cheaper to implement, but more limited in the parameters that they can measure. The choice of system will depend on the specific questions being asked. Passive force systems were first applied to examine skeletal muscle contractility (147), but now there are several passive force system for studying cardiac contractility (121, 148). In a passive force system, the tension in the EHT in between beats gives information about non-sarcomeric contractility and the peak tension in the EHT during contraction gives information about the force of cardiac myosin-driven contractility (Figures 1A,B).

**Figure 2:**
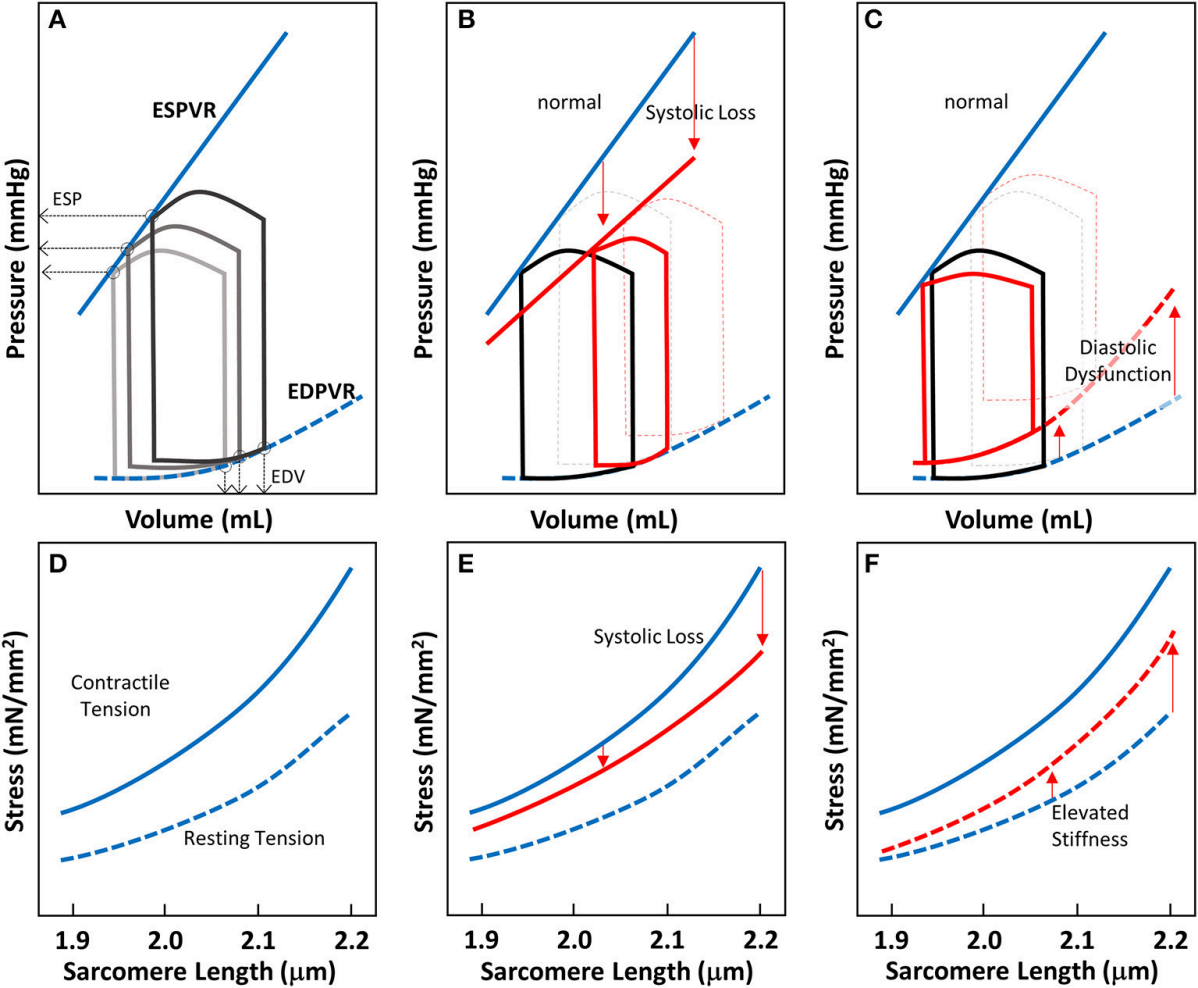
Cardiac pressure-volume (PV)-loop analysis and length-tension relationship of EHTs. **(A)** PV-loops are visualization tools to systematically analyze the contractile properties of heart chamber function. A series of PV-loops can be collected under various preloads (shown in gray). There is a linear relationship between the end systolic pressures (ESP) and their corresponding volume points, known as the end-systolic pressure volume relationship (ESPVR). Similarly, the line connecting the end diastolic volumes (EDVs) and their corresponding pressures is known as the end diastolic pressure volume relationship (EDPVR). **(B)** In systolic heart failure (HFrEF), both the slope of ESPVR and the ejection fraction decrease. **(C)** In diastolic heart failure, the ejection fraction may not change much, but EDPVR shifts upward, indicative of impaired myocardial relaxation during diastole. **(D–F)** Length-tension relationships (LTRs) of EHT strips are related to cardiac functions represented by PV-loops. The contractile tension (solid line) and resting tension (dotted line) are shown. **(D)** LTRs for healthy tissue, showing the contractile and resting stresses. **(E)** In systolic heart failure, there is a reduction in contractile tension that becomes more pronounced as the length is increased (red solid line). **(F)** In diastolic heart failure, the increase in tissue stiffness leads to an increase in resting tension that is more pronounced as the length is increased (red dotted line).

In active force systems, the force on the tissue can be manipulated in real time during an experiment (98, 111, 115, 149–153). This can come from moving one of the posts or from using a probe to manipulate the tissue (Figures 1C,D). Using an active force system, it is possible to examine several important functional properties of the EHT that can be altered in HF (151). In a healthy heart, increasing the stretch of cardiac muscle during diastole causes an increase in cardiac output, an adaptation known as the Frank-Starling relationship. In HF, this relationship is altered, limiting the adaptive capability of the heart. To analyze this relationship, an active force system can stretch the EHT strips with preprogrammed wave forms (Figure 1D) (151). The forces generated during cardiac contraction (i.e., systolic force) and relaxation (i.e., diastolic force) at various tissue lengths are analyzed to generate a cardiac muscle-specific length-tension relationship, LTR (i.e., the Frank-Starling relationship) (Figures 1, 2).

The LTR obtained for EHTs *in vitro* can be related to the work that the heart does *in vivo* during the cardiac cycle. The work done by the whole heart is calculated by measuring pressure-volume (PV) loops during the cardiac cycle (Figure 2A). The work equals the area enclosed within the loop. A family of PV loops can be collected with various preloads to assess cardiac function. As described in Figures 2A–C, one can analyze cardiac function by visualizing the end-systolic pressure-volume relationship (ESPVR) and end-diastolic pressure-volume relationship (EDPVR) at a given inotropic state. For a given stretch/preload, the peak and resting LTR values are related to the ESPVR and EDPVR respectively (Figures 2A,D).

For different types of heart failure, one would expect PV loops to exhibit different ESPVR and EDPVRs. In HFrEF, the loss of systolic function produces a reduced slope of the ESPVR (Figure 2B). To compensate for reduced efficiency of pump function in HFrEF, increasing preload on the heart forces the heart to operate at a higher diastolic volume. In EHTs, the reduction of systolic contractility should appear as a reduction in the contractile force (Figure 2E). The reduction of contractile force should be more pronounced at longer sarcomere lengths (Figure 2E). In HFpEF, there is no change in the ejection fraction, but impaired relaxation due to stiffening of the myocardium. Molecular analysis of the myocardium from patients with HFpEF shows that this elevated passive stiffness can be partly due to stiffening of titin and/or increases in collagen cross-linking (154). As a result of this stiffening, the EDPVR is elevated (Figure 2C). In the EHT strips, HFpEF would be expected to show elevated resting tensions due to this increase in stiffness, and this effect should be more pronounced when the tissue strips are stretched to higher levels of tension (Figure 2F). Taken together, this demonstrates the utility of EHTs for studying cardiac contractility and HF. While this assumption should be validated rigorously, the technology holds promise to be used in HF drug development.

## Application of engineered heart tissues to drug screening

One of the requirements for drug screening is the ability to rapidly screen through large libraries of compounds. The earliest studies of EHT contractility were performed on centimeter scale non-human cardiac tissues in an organ bath (115). In these experiments, over 1 million cells were used to generate a single tissue. The required organ bath was relatively large, requiring 20–50 mL of solution to test a single compound, and it would not be easy to analyze many samples simultaneously for drug screening at this scale. Moreover, the high cost of hPSC culture necessary to generate human tissues makes this system less amenable for drug screens.

To increase assay throughput for drug discovery, various excellent systems have been introduced over the last several years. For example, the Chen lab developed a passive force system where over 100 tissues are formed in microelectromechanical devices in each well of a tissue culture dish (119). Other approaches have focused on fabricating a single EHT in each well of a multi-well plate (e.g., 96/384 well plates) (150, 155). Both of these approaches can be tailored to enable high-throughput screens of libraries of compounds and to provide several physiological readouts of EHT function from a single sample.

As a proof of principle of how EHTs in an active force system can be used for drug screening, we present an example looking at the contractile effects of a drug that is currently in phase III clinical trials as a treatment for systolic heart failure, omecamtiv mecarbil (OM). OM was discovered through a high-throughput screen for compounds that increase cardiac myosin's actin-activated steady-state ATPase activity (156), and OM shows a high affinity for the cardiac myosin isoform (157). OM is a unique positive inotropic compound that was designed to directly activate myosin-based contractility without affecting calcium handling by the cell. This is significant because drugs that target calcium handling can be pro-arrhythmogenic (4). While the exact biophysical mechanism of OM's action on myosin is disputed (28, 158), it has clear positive inotropic effects over a range of dosages (159).

**Figure 3:**
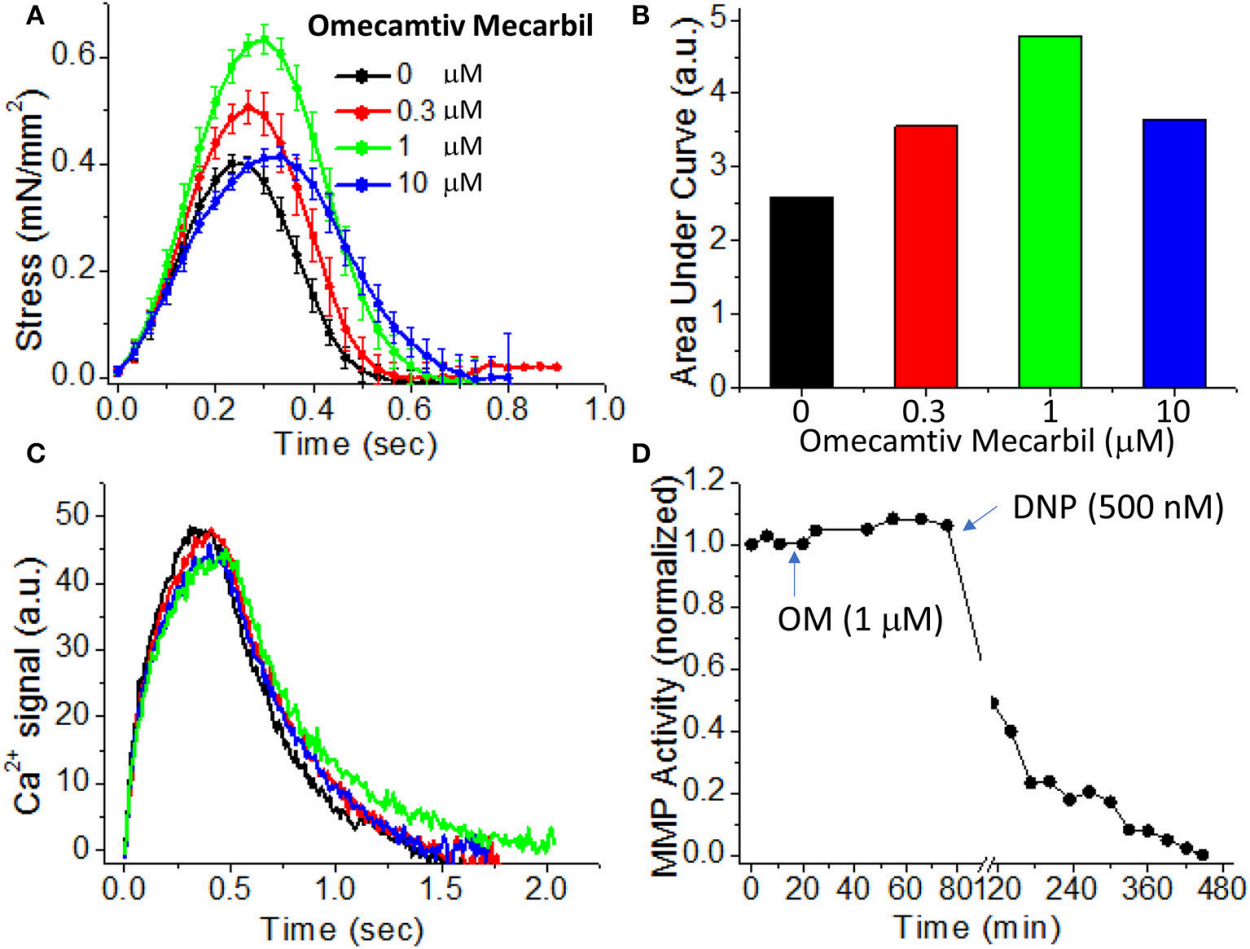
Effects of omecamtiv mecarbil (OM) on excitation-contraction-energy coupling in EHTs. **(A)** EHT strips were exposed to increasing concentrations (0, 0.3, 1, and 10 μM) of OM, and the average time courses of cardiac contraction were analyzed. For OM concentration up to 1 μM, the peak stress increases in a dose-dependent manner. **(B)** The area under each profile in **(A)** was calculated to compare the dose-dependent effects of OM on cardiac contraction profiles. An increase in total contractility was seen with OM treatment. **(C)** Average calcium-transient profiles in the presence of increasing concentrations of OM were measured using a fluorescent calcium dye (*n* = 4–6). OM has little effect on the calcium transient. **(D)** To analyze OM's effects on mitochondrial activity, mitochondrial membrane potential (MMP) activity was monitored for 1 h after compound addition. Treatment with 1 μM OM does not change MMP activity. DNP (2,4-Dinitrophenol), an agent for uncoupling oxidative phosphorylation, was added as a positive control to confirm that the tissue strip was energetically active.

**Figure 4:**
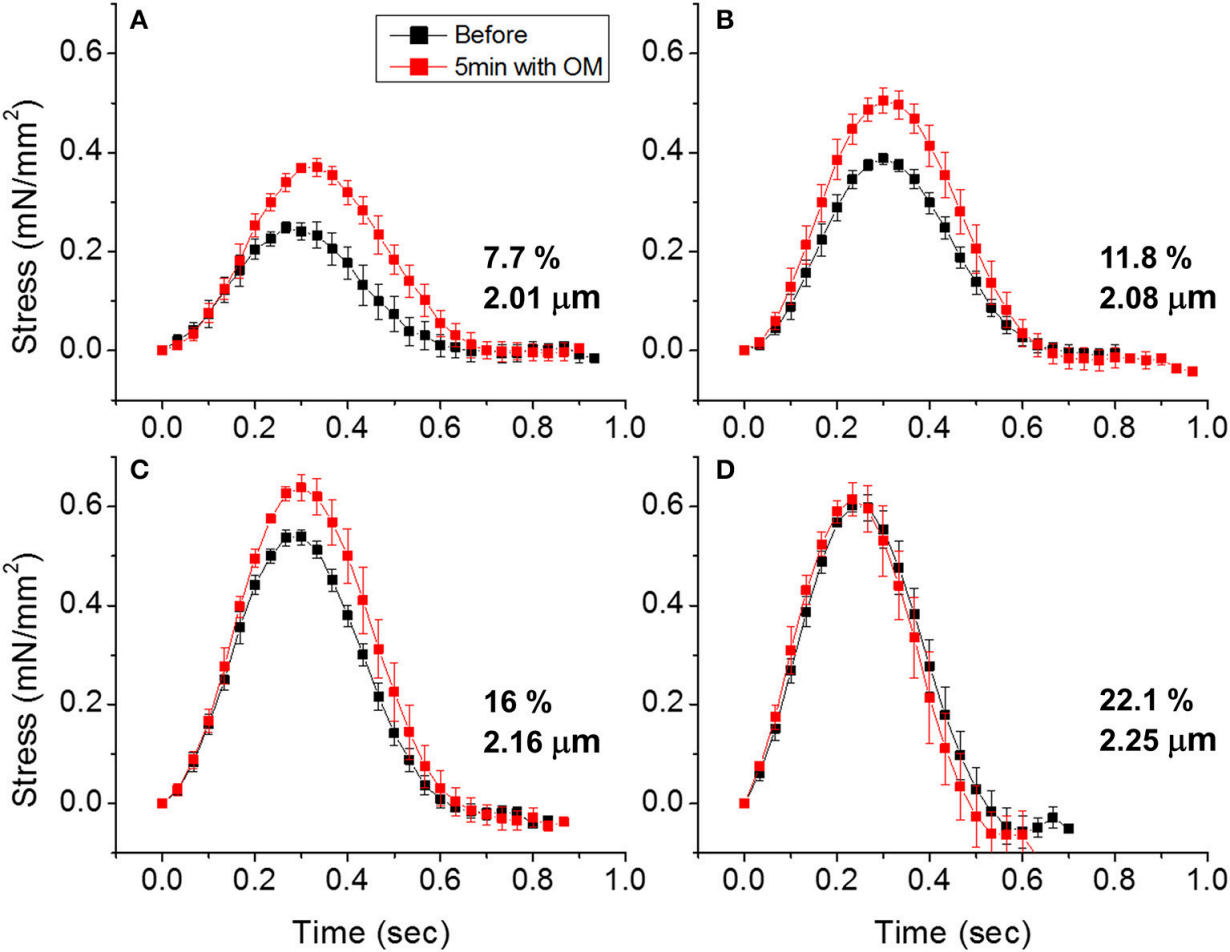
Effects of omecamtiv mecarbil (OM) on LTR in EHTs. **(A)** Cardiac contraction profiles were monitored before and after treating EHT strips with OM (1 μM). At ~2 μm sarcomere length, the OM increased both the peak and duration of cardiac contraction profiles. **(B,C)** As the EHT was stretched further, the effects of OM on contractility compared to the untreated tissue become less pronounced. **(D)** At ~2.25 μm sarcomere length, there is no difference in contractile profile before and after OM treatment.

To demonstrate the effects of OM on EHT contractility, we used an active force system in which stem cell derived EHTs in hydrogels are formed between two parallel bars in each well of a 96-well plate (Figures 1C,D) (150–153, 160). A soft-tissue mechanical analyzer (Palpator, InvivoSciences) measures the mechanical properties of the EHT strips using micro-force transducers attached to its robotic head (150). We first analyzed OM's dose-dependent effects using human EHT strips (Figure 3A). As described previously in rat muscle fibers, active cardiac contractility was increased by concentrations of OM up to 1 μM and inhibited by high concentrations (113). Based on the integrated tension transient (Figure 3B), 1 μM was the most effective concentration tested to increase total contractility. To test the effects of OM on calcium transients, EHT strips were loaded with a biological calcium indicator (Fluo4, Thermo Fisher). As shown in Figure 3C, none of the OM doses tested changed the profiles of calcium transients, consistent with previous reports using non-human cardiomyocytes (28). To analyze OM's effects on metabolic activities, the mitochondrial membrane potential (MMP) was monitored. The mitochondrial activity showed no significant change upon the addition of OM, even with 1 h of incubation. As expected, 2,4-Dinitrophenol (DNP, 500 mM) uncouples the MMP activity. To analyze OM's effects on the LTR, OM (1 μM) was added to EHT tissue strips, and the tissue was stretched to various length using the soft tissue mechanical analyzer (Figure 4). As expected, in the absence of OM, the EHT strips produced increasing levels of cardiac contraction with increasing stretch. After the addition of OM, the tissues produced enhanced contractility with stretch. In general, the tissues generate more stress during a twitch after OM treatment compared to their stress before treatment; however, this difference becomes less pronounced with increasing stretch (Figure 4D). These results demonstrate the ability to use hPSC-CM EHT systems for drug testing. When combined with gene edited cells, this approach will open the door for targeted therapeutic design and precision medicine.

## Future prospects for drug development and precision medicine

Even though deaths from cardiovascular disease accounted for >20% of all deaths in the US, cardiovascular drugs account for only 6.6% of compounds in Phase I clinical trials that were eventually approved for patient use (161). One of the difficulties with developing new cardiovascular treatments is the huge cost of clinical trials, which require large study cohorts to evaluate the efficacy of treatments for chronic diseases, such as age-associated HF. A recent analysis of 9,985 clinical and regulatory phase transition records between 2006 and 2015 indicates that the likelihood of approval increases three-fold when working with a targeted, well-defined patient population (161). The use of reliable biomarkers to select patients and monitor their responses has been shown to improve the performance of treatment candidates during trials. The use of genetically engineered cells in EHTs should allow for the development of preclinical disease models that mimic heart failure against a controlled genetic background.

The combined use of genetic engineering and tissue engineering can be used to model monogenic cardiac disease *in vitro* as well as the role of genetic modifiers in disease. Importantly, these tools can be harnessed for precision medicine. For example, one critical bottleneck in the treatment of genetic heart disease is identifying whether a given genetic variant identified in a patient is pathogenic or not. We envision that EHTs generated from genetically engineered cells will enable the direct testing of the consequences of specific genetic variants. Moreover, the use of reprogrammed cells taken from a patient cheek scraping or urine sample will open the door to the development of personalized medicine. EHTs generated from these cells can be used to evaluate the efficacy and side effects of precision therapies, enabling clinicians to optimize the treatment course for each patient. These applications will be aided by high-throughput EHT phenotyping. Taken together, these advances have the potential to revolutionize the treatment of cardiac disease.

## Author contributions

MG and TW wrote and edited the manuscript. ND, MC, AW, and TW designed and performed experiments, analyzed data, and created figures.

### Conflict of interest statement

MG's contributions were conducted in the absence of any commercial or financial relationships that could be construed as a potential conflict of interest. ND, MC, AW, and TW are employees of a for-profit organization, InvivoSciences, Inc.
